# Role of resistin as a marker of inflammation in systemic lupus erythematosus

**DOI:** 10.1186/ar2366

**Published:** 2008-01-30

**Authors:** Katarina Almehed, Helena Forsblad d'Elia, Maria Bokarewa, Hans Carlsten

**Affiliations:** 1 Department of Rheumatology and Inflammation Research, Sahlgrenska Academy at Göteborg University Guldhedsgatan 10, S-413 46 Göteborg, Sweden

## Abstract

**Introduction:**

Resistin is a cystein-rich secretory adipokine. It is proposed to have proinflammatory properties in humans. The aim of this study was to determine associations between serum levels of resistin and markers of inflammation and bone mineral density (BMD) in female patients with systemic lupus erythematosus (SLE).

**Methods:**

One hundred sixty-three female patients with SLE (20 to 82 years old) were examined in a cross-sectional study. Venous blood samples were analyzed for resistin, erythrocyte sedimentation rate (ESR), C-reactive protein, creatinine, fasting lipids, complements, tumor necrosis factor-alpha, interleukin (IL)-1β, IL-6, sIL-6R (soluble IL-6 receptor), ICTP (C-terminal telopeptide of type I collagen), and PINP (N-terminal propeptide of type I procollagen). Simple and multiple regression analyses as well as logistic regression analyses were performed. Resistin in serum was compared with 42 healthy female controls with respect to age.

**Results:**

Serum resistin levels in controls were similar to those of patients with SLE. Markers of inflammation and current dose of glucocorticosteroids correlated positively to resistin in serum. Markers of renal function, number of prevalent vertebral fractures, and BMD were also significantly associated with resistin. In a multiple regression model, ESR, creatinine, C3, current glucocorticosteroid dose, high-density lipoprotein, and BMD radius remained significantly associated with resistin. In logistic regression analyses with resistin as the independent variable, a significant association was found with ESR (normal or elevated) but not with S-creatinine or *z *score for hip and radius total.

**Conclusion:**

Although resistin measurements did not differ between patients and controls, resistin was clearly associated with general inflammation, renal disease, treatment with glucocorticosteroids, and bone loss. We hypothesize that resistin has proinflammatory and disease-promoting properties in SLE. Further studies are needed to elucidate the mechanism behind these associations.

## Introduction

Resistin is a recently described, low-molecular-weight, cystein-rich secretory peptide [1-3]. It is also known as adipocyte-specific secretory factor. Animal studies show that resistin is produced mainly in white adipose tissue and may be the linkage between obesity and insulin resistance. In humans, the role of resistin is not yet fully established. There is evidence that resistin has proinflammatory properties and is abundant in inflammatory diseases (for instance, rheumatoid arthritis (RA) [4] and Crohn disease [5]) and also is associated with inflammatory markers in several different populations [6-8]. In humans, resistin is expressed in inflammatory cells, leukocytes, and macrophages [9] and has the potency of inducing production of interleukin (IL)-6 and tumor necrosis factor-alpha (TNF-α) [9,10]. Resistin is accumulated in inflamed joints of patients with RA and has the capacity to induce arthritis in mice [11]. There are also data indicating that resistin levels are inversely associated with renal function and possibly contribute to a low-grade inflammation in patients with chronic renal dysfunction [12]. Resistin seems to be of importance in bone metabolism, stimulating osteoblast and osteoclast differentiation, possibly mediated directly or indirectly through the nuclear factor kappa B (NF-κB) pathway [13]. Systemic lupus erythematosus (SLE) is a disease characterized by systemic inflammation with the property of affecting several organs throughout the body, including kidneys. Therefore, we wanted to examine the relationship and possible associations between resistin and different markers of disease activity, inflammation, renal function, lipids, and bone mineral density (BMD) in a female cohort of patients with SLE.

## Materials and methods

### Patients

All patients with SLE treated in the rheumatology clinics in Göteborg and Borås, in western Sweden, were identified from administrative registers and invited to participate in this cross-sectional study. The procedure of enrollment has been described in detail [14]. In short, 339 patients (298 women and 41 men) were identified. There was a 70% reply frequency among the female patients. One hundred sixty-three female patients fulfilling at least four of the 1982 American College of Rheumatology (ACR) classification criteria for SLE [15] were included in and completed the study. Only data regarding female patents have been analyzed. For each patient, data on age, duration of disease, weight, and height were recorded. Medication, smoking habits, physical activity, and clinical fractures were assessed by self-administered questionnaires. The Systemic Lupus Erythematosus Disease Activity Index (SLEDAI-2K) [16] was used to score disease activity. Disease damage was recorded according to the Systemic Lupus International Collaborative Clinics/ACR Damage Index [17]. Glomerular filtration rate (GFR) was predicted using the Cockcroft and Gault equation [18]. GFR (mL/minute) = (140 - age) × weight (kg) × 1.04/S-creatinine (μmol/L). Cumulative corticosteroid intake was calculated by reading the medical records of all patients. The same rheumatologist assessed all patients (KA).

### Laboratory tests

Venous blood samples were taken after a one-night fast. Serum from the venous blood samples was stored at -70°C until the time of analyses. However, erythrocyte sedimentation rate (ESR), C-reactive protein (CRP), blood cell count, creatinine, C3, C4, and the plasma lipoproteins, total cholesterol, high-density lipoprotein (HDL), low-density lipoprotein, and triglycerides (Tg) were analyzed consecutively using standard laboratory techniques in the Department of Clinical Chemistry of Sahlgrenska University Hospital.

### Bone markers

The bone resorption marker, C-terminal telopeptide of type I collagen (ICTP), and the bone formation marker, N-terminal propeptide of type I procollagen (PINP), were analyzed quantitatively in serum by radioimmunoassay (Orion Diagnostica, Espoo, Finland). Detection limits were ICTP 0.7 μg/L and PINP 2 μg/L.

### Resistin

Resistin levels were detected with a sandwich enzyme-linked immunosorbent assay (ELISA) (R&D Systems, Inc., Minneapolis, MN, USA). Briefly, samples diluted 1:10 with 1% bovine serum albumin phosphate-buffered saline were introduced into the parallel strips coated with capture polyclonal anti-resistin antibodies. Biotin-labelled anti-resistin antibodies, streptavidin-horseradish peroxidase conjugate, and corresponding substrate were used for color development. The obtained absorbance values were compared with the serial dilution of recombinant human resistin. The lowest detectable level was 31 pg/mL.

### Cytokines

Quantitative sandwich ELISA kits were used for measurement of proinflammatory cytokines TNF-α, IL-1β, IL-6, and soluble IL-6 receptor (sIL-6R) (Quantikine; R&D Systems, Inc.) with detection limits of 0.12, 0.1, 0.7, and 6.5 pg/mL, respectively.

### Bone mineral density measurements

Lumbar spine (L2–L4), non-dominant hip (femoral neck and total hip), and non-dominant distal forearm were measured by DXA (dual-energy x-ray absorptiometry) with a Lunar Prodigy densitometer 12165 (GE Healthcare, Little Chalfont, Buckinghamshire, UK). The precisions for duplicate measurements were 0.9% for lumbar spine, 0.5% for left total hip and femoral neck, and 2.8% for radius. All BMD results were expressed in absolute values (g/cm^2^) and as the number of standard deviations (SDs) above or below the mean results of age-matched women (*z *score).

### Fractures

Lateral x-rays of thoracic and lumbar spine (Th4-L4) were evaluated for prevalent vertebral compression fractures by a visual semiquantitative method (the method of Genant and colleagues [19]). All vertebral deformities of at least 20% to 25% reduction of height, anterior, middle and/or dorsal were regarded as compression fractures. One radiologist performed all analyses.

### Healthy controls

A control group of 12 female healthy blood donors and 30 healthy female staff members and PhD students in the Department of Rheumatology were analyzed for serum levels of resistin.

### Ethical considerations

All patients gave informed written consent prior to participation, and the study was approved by the ethics committee at Sahlgrenska Academy at Göteborg University.

### Statistical analysis

Analyses were performed using SPSS version 12.0.1 (SPSS Inc., Chicago, IL, USA). Descriptive statistics are presented as median and range or as mean and SD. All variables were tested with the Kolmogorov-Smirnov normality test. Pearson correlation was used when the variables were normally distributed; otherwise, Spearman correlation was used. Significant variables were then entered in the multiple linear regression analyses as independent variables and resistin as a dependent variable. A forward stepwise method was used.

ESR and S-creatinine were defined as normal or pathological according to standard laboratory normal values. These variables were dependent in a logistic forward regression analyses with resistin as the independent variable. The same method was used for *z *score hip total and radius total with the cutoff value of -1 SD. A receiver operating characteristic (ROC) curve was then calculated with ESR (elevated or not), S-creatinine (elevated or not), *z *score hip total, and *z *score radius total (cutoff value of -1 SD) as the state variable and resistin as the test variable. The constant and the regression coefficients of patients with SLE were compared with controls with respect to resistin and age by means of a special *t *test. All tests were two-tailed, and a *p *value of less than 0.05 was considered statistically significant.

## Results

### Demographic and disease-related variables

The SLE patients participating in this study did not differ significantly in age from those who were invited but did not participate. The general characteristics of the study population are presented in Table 1. The participants' ages ranged from 20 to 82 years. Seventy-two (44%) women were premenopausal. Ninety-one (56%) were on disease-modifying antirheumatic drugs, and 85 (52%) were treated with glucocorticosteroids. Only one person had end-stage renal disease, but 4 (2.5%) had impaired renal function with a GFR of less than 30 mL/minute, and 23 (14%) had a GFR of less than 50 mL/minute by use of the predicted GFR value.

**Table 1:** Demographic and disease-related variables in 163 female patients with systemic lupus erythematosus

Demographic variables	Value
Patient age, years	47 (20 to 82)
Weight, kg	66 (42 to 99)
Height, cm	166 (145 to 182)
Body mass index, kg/m^2^	24.2 (17.2 to 37.2)
Menopausal status	
Premenopausal, n (%)	72 (44)
	
Disease variables	
Disease duration, years	11 (1 to 41)
SLEDAI-2K	5 (0 to 31)
SLICC/ACR Damage Index	2 (0 to 11)
Kidney affection ever by SLE, n (%)	40 (25)
S-creatinine, μmol/L	87 (49 to 291)
Glomerular filtration rate, mL/minute	74 (22 to 172)
Proteinuria, >3.5 g/24 hours, n (%)	9 (6)
End-stage kidney disease, n (%)	1 (0.6)
	
Hemoglobin, g/L	131 (75 to 158)
Erythrocyte sedimentation rate, mm/hour	25 (2 to 125)
C-reactive protein, mg/L	5 (3 to 100)
Cholesterol, mmol/L	5.4 (2.4 to 9.3)
High-density lipoprotein, mmol/L	1.6 (0.5 to 2.8)
Low-density lipoprotein, mmol/L	3.1 (<0.1 to 6.3)
Triglycerides, mmol/L	1.2 (0.3 to 6.0)
Albumin, g/L	40 (11 to 53)
IgG, g/L	13.5 (5 to 28)
IgA, g/L	2.5 (0.07 to 11)
IgM, g/L	1.0 (0.05 to 4.6)
C3, g/L	0.93 (0.28 to 1.68)
C4, g/L	0.14 (0.02 to 0.28)
Tumor necrosis factor-alpha, pg/mL	2.16 (0.40 to 36.96)
Interleukin-1β, pg/mL	0.47 (0.0 to 9.65)
Interleukin-6, pg/mL	9.67 (2.81 to 119.0)
sIL-6R, ng/mL	48.87 (11.56 to 107.15)
	
Glucocortocosteroid user, n (%)	85 (52)
Glucocorticosteroid dose, mg	5 (2.5 to 35)
	
BMD lumbal spine, g/cm^2^, mean (SD)	1.12 (0.18)
BMD total hip, g/cm^2^, mean (SD)	0.92 (0.15)
BMD femur neck, g/cm^2^, mean (SD)	0.89 (0.15)
BMD radius total, g/cm^2^, mean (SD)	0.50 (0.08)
Number of vertebral fractures per patient	0 (0 to 11)
ICTP, μg/L	3.59 (0.9 to 16.38)
PINP, μg/L	43.0 (9.1 to 177.94)

Values are presented as median (and range) unless indicated otherwise. BMD, bone mineral density; ICTP, C-terminal telopeptide of type I collagen; Ig, immunoglobulin; PINP, N-terminal propeptide of type I procollagen; SD, standard deviation; sIL-6R, soluble interleukin-6 receptor; SLE, systemic lupus erythematosus; SLEDAI, Systemic Lupus Erythematosus Disease Activity Index; SLICC/ACR, Systemic Lupus International Collaborative Clinics/American College of Rheumatology.

### Resistin and associated factors

The median serum resistin level was 6.53 (2.23 to 19.14) ng/mL. Several clinical and laboratory disease-related variables were significantly associated with resistin levels in serum using a simple regression model (Table 2). Markers of inflammation in SLE such as raised ESR, CRP, immunoglobulin G (IgG), proinflammatory cytokines, and low S-albumin levels correlated to resistin in serum. There was also an association between resistin and impaired renal function and current dose of corticosteroids. Bone variables such as number of vertebral fractures, low BMD in three of four measured sites, and the bone resorption marker ICTP also correlated to high resistin. Tg values were positively associated with resistin, whereas HDL was inversely associated with resistin. A multiple regression model, with resistin as the dependent variable and with the variables significantly correlated to resistin (Table 2) as independent variables, was performed. High inflammation, impaired renal function, medication with glucocorticosteroids, high HDL, and low BMD in radius remained significant markers of high resistin levels (Table 3).

**Table 2:** Correlation coefficients (r) of resistin (dependent variable) and disease-related variables (independent variables).

	Resistin (ng/mL)	
	r	r_s_

Erythrocyte sedimentation rate, mm/hour		0.316^a^
C-reactive protein, mg/L		0.193^c^
S-albumin, g/L		-0.302^a^
IgG, g/L		0.178^c^
S-C3, g/L	-0.216^b^	
Interleukin-6, pg/mL		0.315^a^
sIL-6R, pg/mL	0.183^c^	
Tumor necrosis factor-alpha, pg/mL		0.312^a^
		
S-high-density lipoprotein, mmol/L	-0.177^c^	
S-triglycerides, mmol/L		0.252^b^
		
S-creatinine, μmol/L		0.180^c^
Glomerular filtration rate, mL/minute	-0.228^b^	
Nephritis ever (yes = 1 and no = 0)		0.178^c^
Corticosteroid current dose, mg/day		0.157^c^
		
BMD lumbar spine, g/cm^2^	-0.165^c^	
BMD total hip, g/cm^2^		-0.170^c^
BMD radius total, g/cm^2^		-0.261^b^
Number of vertebral fractures per patient		-0.171^c^
S-ICTP, μg/L		0.193^c^

All variables were tested with the Kolmogorov-Smirnov normality test. Pearson correlation was used when the variables were normally distributed; otherwise, Spearman correlation was used. Only significant variables are shown. ^a^*p *< 0.001; ^b^*p *< 0.01; ^c^*p *< 0.05. BMD, bone mineral density; ICTP, C-terminal telopeptide of type I collagen; IgG, immunoglobulin G; r, Pearson correlation coefficient; r_s_, Spearman correlation coefficient; sIL-6R, soluble interleukin-6 receptor; SLE, systemic lupus erythematosus.

**Table 3:** Multiple stepwise regression analysis of resistin (dependent variable) and demographic and disease-related variables (independent variables)

	Resistin (ng/mL)		
	11.155		

	β	SE	*P *value

Erythrocyte sedimentation rate, mm/hour	0.044	0.012	0.001
S-creatinine, μmol/L	0.035	0.008	<0.001
Complement factor C3, g/L	-2.915	1.023	0.005
Glucocorticosteroid current dose, mg/day	0.127	0.051	0.014
High-density lipoprotein, mmol/L	-1.438	0.580	0.014
Bone mineral density radius, g/cm^2^	-7.133	3.156	0.026
			
*R^2^*	0.42		

The regression equation of resistin (ng/mL) = 11.155 + 0.044 × erythrocyte sedimentation rate (mm/hour) + 0.035 × S-creatinine (μmol/L) - 2.915 × C3 (g/L) + 0.127 × current steroid dose (mg/day) - 1.438 × high-density lipoprotein (mmol/L) - 7.133 × bone mineral density radius (g/cm^2^). Beta values are unstandardized regression coefficients. *R*^2 ^is equal to the variance explained in the model. SE, standard error.

Logistic forward regression analyses were performed with resistin as the independent variable and normal or pathological ESR or S-creatinine as dependent variables. Analyses were also performed with *z *score total hip and radius as dependent variables using a cutoff value as -1 SD for normal or reduced bone mass. Resistin was significantly associated with ESR but not with S-creatinine, *z *score hip total, or *z *score radius total (Table 4).

**Table 4:** Resistin as independent variable in logistic regressions and test variable in area under ROC curves

	Logistic regression with resistin as independent variable^a^	ROC curve with resistin as test variable^b^
	*P *value	Area under ROC curve (95% CI)

Erythrocyte sedimentation rate	0.001	0.66 (0.58 to 0.75)
S-creatinine	0.19	0.55 (0.46 to 0.65)
*Z *score hip total	0.2	0.57 (0.46 to 0.67)
*Z *score radius total	0.13	0.56 (0.46 to 0.66)

^a^Erythrocyte sedimentation rate (ESR) and S-creatinine were defined as normal or pathological according to standard laboratory normal values. These variables were dependent in logistic conditional forward regression analyses with resistin as independent variable. The same method was used for *z *score hip total and radius total with the cutoff value of -1 standard deviation (SD). ^b^Area under the receiver operating characteristic (ROC) curve was calculated with ESR (normal or not), S-creatinine (normal or not), *z *score hip total, and *z *score radius total (above or below -1 SD) as the state variable and resistin as test variable. Null hypothesis: true area under ROC curve = 0.5. CI, confidence interval.

### Resistin in patients with systemic lupus erythematosus compared with controls

**Figure 1:**
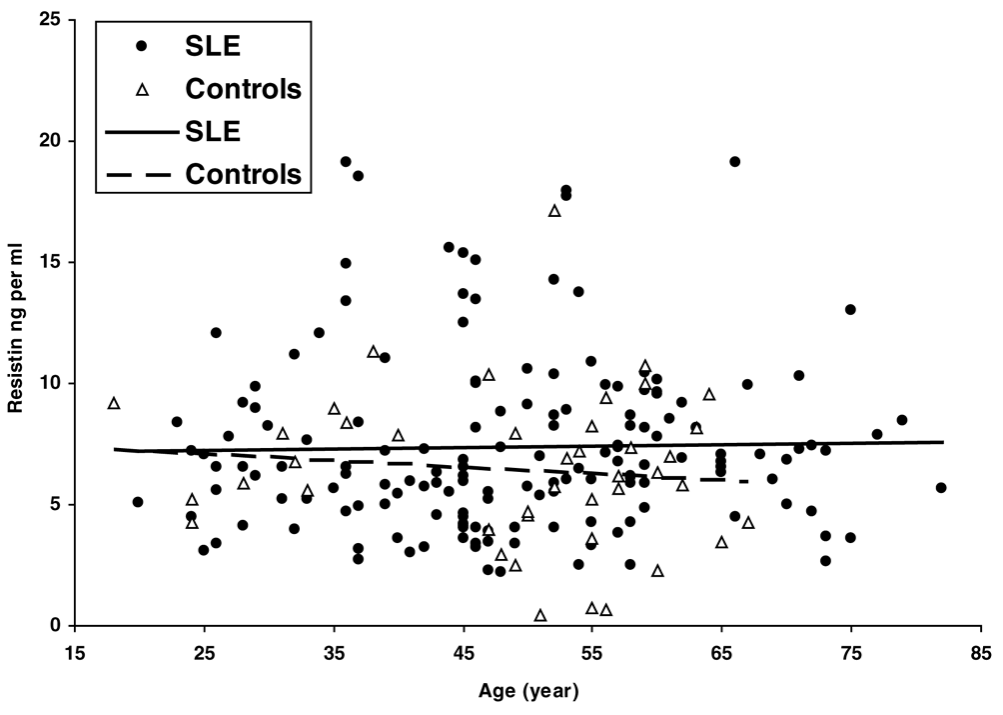
Correlation between serum resistin levels and age in systemic lupus erythematosus (SLE) patients and controls. The constant and the regression coefficients, with respect to resistin values and age, were compared with the corresponding parameters of the controls by use of a special *t *test. The regression coefficient is the slope. No significant difference was found between these parameters.

Forty-two healthy controls with a median age of 52 (18 to 67) years had a median serum resistin value of 6.24 (0.47 to 17.12) ng/mL. The constant and the regression coefficients of the patients with SLE, with respect to resistin values and age, were compared versus the corresponding parameters of the controls by use of a special *t *test. No significant difference was found between the patients with SLE and the controls (Figure 1).

## Discussion

Resistin is an adipokine and a novel cytokine with proinflammatory properties in humans. To our knowledge, this is the first time resistin has been analyzed in the serum of a large cohort of patients with SLE. Our results indicate a clear association between resistin and inflammation, complement levels, BMD, and renal function in SLE. It is too early to assess resistin as a pathogenic factor in SLE disease, although the associations of resistin with low complement levels and the apparent central position in the proinflammatory cytokine cascade make it an interesting subject for further investigation.

In this cross-sectional study of female patients with SLE, resistin was positively associated with inflammation even though resistin levels were not significantly increased compared with controls. Resistin exerts its main action locally in different tissue departments, and the measured serum levels may reflect only a small spillover into the blood compartment [11].

Inflammation in SLE is in contrast to inflammation in other rheumatic diseases characterized by elevated ESR while CRP often remains low. In spite of this, there were associations between resistin and CRP (*r *= 0.193) as well as between resistin and ESR (*r *= 0.316). Severe SLE flares (for example, in kidneys and skin) are known to be immune-complex-mediated and accompanied by complement activation and consumption. In our material, resistin correlated to low C3 and high IgG as well as to elevated proinflammatory cytokines such as IL-1β, IL-6, sIL-6R, and TNF-α. The low serum albumin reflects inflammation, but in the case of nephrotic syndrome in 9 (6%) patients, large renal loss of proteins could affect the simple regression outcome. In multiple regression analyses, with resistin as the dependent variable, ESR and low C3 remained significant markers of high resistin levels. Our interpretation is that resistin acts as a marker both of general inflammation exemplified by ESR and of SLE-specific immune-complex-mediated disease activity exemplified by low C3. When ESR was used as the dependent variable in logistic regression analyses (elevated ESR or not), resistin was also significantly associated with ESR (area under the ROC curve = 0.66). In comparison with this result, one may refer to an investigation showing a similar connection, in which the risk of peripheral arterial disease in type 2 diabetes mellitus when HbA1c increased 1 SD generated an area under the ROC curve of 0.64 [20].

An association between resistin and inflammation has been reported in several different diseases, including RA [21] and inflammatory bowel disease [5], but is very weak or nonexistent in studies of apparently healthy individuals [22]. We found that current glucocorticosteroid dose correlated positively to resistin levels and remained a significant variable of resistin in multiple regression analyses. Resistin production in mouse adipocytes has been shown to increase after exposure to dexamethasone [23]. In a patient population, however, it is difficult, if not impossible, to separate the effect of steroid medication by itself from the disease activity it is meant to influence.

The relationship between obesity and expression of resistin is not clear in humans, although the transcription of resistin mRNA is high in preadipocytes during differentiation. Resistin has been shown to correlate to low HDL in a cross-sectional Japanese population [24] and to low HDL and high Tg in a European general population [25]. In rheumatic diseases, dyslipoproteinemia is seen and is also known to be linked to inflammation in SLE [26-28] and possibly also to the use of glucocorticosteroids [29]. We found that resistin was associated with high Tg and low HDL but not with total cholesterol, weight, or body mass index. HDL was significantly associated with resistin in the multiple regression model. If resistin acts as a proinflammatory molecule, it could be one important link in the intricate interactions between inflammation and dyslipoproteinemia and subsequently atherosclerosis seen in SLE and other inflammatory conditions [30,31].

We demonstrated a positive correlation between resistin, creatinine, and ever having had nephritis and a negative correlation between resistin and GFR. Similar associations have been shown in different patient groups (for example, in patients with coronary heart disease [32], in kidney allograft recipients [7], and in a small number of children with end-stage renal disease [33]). Yaturu and colleagues [12] found significantly higher resistin levels in patients with chronic kidney disease compared with controls but no correlation to GFR. Several of the mentioned reports also revealed a correlation between resistin and inflammation. In our study, serum levels of creatinine remained a significant variable to resistin in the multiple regression analyses. Whether this is due to high systemic inflammation in the patients having ongoing lupus nephritis or to resistin merely being accumulated in the serum of patients with low GFR cannot be decided. Resistin has been shown to be a regulator and to increase the release of IL-1β, IL-6, and TNF-α in human peripheral blood mononuclear cells via the NF-κB pathway. Several endogenous substances like proinflammatory cytokines have also been shown to upregulate resistin gene expression [11,34]. The NF-κB pathway is involved in osteoclastogenesis, and resistin has been found to stimulate osteoclast differentiation from human peripheral monocytes and, to a lesser extent, osteoblast proliferation in humans [13]. Several studies indicate a more pronounced development of osteopenia and osteoporosis in patients with SLE than in controls, and not only due to the use of glucocorticosteroids [14]. Therefore, it was interesting that BMD in three of four measured locations and the number of radiological vertebral compression fractures correlated inversely to resistin. The bone resorption marker ICTP correlated positively to resistin. In multiple regression analyses, only BMD in radius remained associated with resistin. Oh and colleagues [35] have shown an inverse correlation of resistin to BMD in lumbar spine in an adult male Korean patient cohort also indicating the connection between resistin and bone metabolism.

## Conclusion

In patients with SLE, we now show a clear association between resistin and inflammation, impaired kidney function, low complement levels, use of glucocorticosteroids, BMD, and low HDL. Whether resistin has pathophysiological significance in SLE or whether it should be regarded solely as a marker of inflammation is, for the moment, impossible to say. We encourage and look forward to both clinical and mechanistical studies in this field.

## Abbreviations

ACR = American College of Rheumatology; BMD = bone mineral density; CRP = C-reactive protein; ELISA = enzyme-linked immunosorbent assay; ESR = erythrocyte sedimentation rate; GFR = glomerular filtration rate; HDL = high-density lipoprotein; ICTP = C-terminal telopeptide of type I collagen; IgG = immunoglobulin G; IL = interleukin; NF-κB = nuclear factor kappa B; PINP = N-terminal propeptide of type I procollagen; RA = rheumatoid arthritis; ROC = receiver operating characteristic; SD = standard deviation; sIL-6R = soluble interleukin-6 receptor; SLE = systemic lupus erythematosus; Tg = triglycerides; TNF-α = tumor necrosis factor-alpha.

## Competing interests

The authors declare that they have no competing interests.

## Authors' contributions

KA conceived the study, participated in its design and coordination, performed most of the statistical analyses, and drafted the manuscript. HFd'E participated in study design and coordination, interpretation of statistical analyses, and revision of the manuscript. MB contributed with samples from controls, contributed important knowledge about resistin, and participated in revision of the manuscript. HC participated in study design, interpretation of data, and revision of the manuscript. All authors read and approved the final manuscript.
